# Regional brain dysfunction in insomnia after ischemic stroke: A resting-state fMRI study

**DOI:** 10.3389/fneur.2022.1025174

**Published:** 2022-11-25

**Authors:** Hongzhuo Wang, Yunxuan Huang, Mingrui Li, Han Yang, Jie An, Xi Leng, Danghan Xu, Shijun Qiu

**Affiliations:** ^1^Medical Imaging Center, The First Affiliated Hospital of Guangzhou University of Traditional Chinese Medicine, Guangzhou, China; ^2^Rehabilitation and Nursing Center, The First Affiliated Hospital of Guangzhou University of Chinese Medicine, Guangzhou, China; ^3^Department of Magnetic Resonance Imaging, Zhanjiang First Hospital of Traditional Chinese Medicine, Zhanjiang, China

**Keywords:** post-stroke insomnia, functional magnetic resonance imaging, regional homogeneity, amplitude of low-frequency fluctuations, fractional amplitude of low-frequency fluctuation

## Abstract

**Objective:**

This study aimed to explore the abnormality of local brain function in patients with post-stroke insomnia (PSI) based on fMRI and explore the possible neuropathological mechanisms of insomnia in patients with PSI in combination with the Pittsburgh sleep quality index (PSQI) score and provide an objective evaluation index for the follow-up study of acupuncture treatment of PSI.

**Methods:**

A total of 27 patients with insomnia after stroke were enrolled, and the PSQI was used to evaluate their sleep status. Twenty-seven healthy participants who underwent physical examinations during the same period were selected as controls. Resting-state brain function images and structural images of the two groups of participants were collected, and the abnormal changes in the regional brain function in patients with PSI were analyzed using three methods: regional homogeneity (ReHo), the amplitude of low-frequency fluctuations (ALFF) and fractional ALFF (fALFF), and a correlation analysis with the PSQI scale score.

**Results:**

Compared with the HCs, the ReHo values of the PSI group in the bilateral lingual gyrus, right cuneus, right precentral and postcentral gyri were significantly lower, and the ReHo values of the left supramarginal gyrus were significantly higher. In the PSI group, the ALFF values in the bilateral lingual gyrus were significantly decreased, whereas those in the bilateral middle temporal gyrus, right inferior temporal gyrus, right inferior frontal gyrus, right limbic lobe, right precuneus, left posterior cingulate gyrus, and left middle occipital gyrus were significantly increased. Compared with HCs, the fALFF values of the bilateral lingual gyrus, bilateral inferior occipital gyrus, and bilateral cuneus in the PSI group were significantly higher. The ReHo value of the left supramarginal gyrus in the PSI group was significantly negatively correlated with the total PSQI score.

**Conclusion:**

Patients with PSI have abnormal local activities in multiple brain regions, including the visual processing-related cortex, sensorimotor cortex, and some default-mode network (DMN) regions. Over-arousal of the DMN and over-sensitivity of the audiovisual stimuli in patients with PSI may be the main mechanisms of insomnia and can lead to a decline in cognitive function and abnormalities in emotion regulation simultaneously.

## Introduction

Post-stroke insomnia (PSI) refers to insomnia symptoms that occur after a stroke, and the rate of sleep disturbance in post-stroke patients is approximately 30–48% (1). As many as 70% of acute stroke patients have associated sleep disorders, mainly manifested as difficulty in falling asleep at night, difficulty in maintaining sleep or awakening early, difficulty in falling asleep after awakening, and daytime sleepiness and fatigue (2). Sleep disturbance in patients with PSI is highly detrimental to stroke recovery and contributes to the deterioration of existing diseases, such as hypertension and diabetes, reducing patients' quality of life (3). Current studies suggest that the mechanisms of insomnia in patients with PSI are as follows: the stroke lesions in patients with PSI involve important brain regions, such as the thalamus and the basal ganglia, and the damage to these regional brain tissues leads to the blockage of transmission pathways of neurotransmitters such as 5-hydroxytryptamine and norepinephrine in the brain (4); or the reduction of gamma-aminobutyric acid content in the serum of patients with stroke leads to the reduction of neuronal activity that promotes sleep (5, 6); or the interaction between negative emotions, such as anxiety, after stroke and 5-hydroxytryptamine eventually leads to insomnia (7, 8). All these alterations lead to abnormalities in brain function, resulting in insomnia symptoms; however, the specific mechanism of PSI is still unclear.

Resting-state functional magnetic resonance (rs-fMRI) measures spontaneous low-frequency fluctuations in brain activity through blood oxygen level-dependent (BOLD) signals (9, 10), which can not only reveal the intrinsic function of the brain in healthy participants but also identify disease-state changes in the intrinsic functional connectivity (FC) of the brain (11–13) and reveal the brain mechanism of neuromodulation in the treatment of clinical diseases (14–16). Regional homogeneity (ReHo) (17) and amplitude of low-frequency fluctuations (ALFF) are two important indicators of rs-fMRI to measure regional brain functional activity. Their post-processing techniques are mature, and the results are stable and reliable (18, 19). Multiple studies (20–22) using ReHo analysis in patients with primary chronic insomnia have consistently found that ReHo values of the left cuneus and left parahippocampal gyrus are significantly increased, while activity abnormalities in other brain regions show variable findings in different studies, some studies even presented conflicting results. Most studies (20, 22) have shown that ReHo values change in brain regions related to emotion and cognition, negative emotions, and abnormal emotional regulation in patients with primary chronic insomnia lead to insomnia symptoms, but the mechanism remains unclear. However, studies (23, 24) using ALFF analysis in patients with primary chronic insomnia have found that the ALFF values are elevated in the temporal and occipital lobes and decreased in the prefrontal lobe and cerebellar hemispheres, which may be associated with cognitive impairment and hyperactive audiovisual responses, but further research is needed for confirmation.

We hypothesized that patients with PSI have similar arousal mechanisms with patients with primary chronic insomnia, and their abnormal brain functional activities may be related to their cognition and emotion regulation. We used rs-fMRI to explore abnormal changes in local brain function in patients with PSI to provide a basis for further research on the neuropathological mechanism of insomnia in patients with PSI.

## Materials and methods

### Participants

A total of 27 patients with insomnia after stroke were included in the PSI group, and 27 healthy controls (HCs) matched for age, sex, and years of education were enrolled in this study. The quality of sleep for all participants with PSI was assessed using the Pittsburgh Sleep Quality Index (PSQI) (25), which was not evaluated in the HCs.

The inclusion criteria for the PSI group were as follows: (1) All patients with stroke had ischemic stroke and the stroke lesion was diagnosed using brain MRI in the acute phase; the history of stroke was ≥ 6 months; the infarct location was confined to the deep white matter or basal ganglia, thalamus, brainstem. The lesion length in the luminal infarct was ≤ 10 mm, and the number of lesions in every patient was not limited. (2) Insomnia symptoms were secondary to stroke and fulfilled the fifth edition of the Diagnostic and Statistical Manual of Mental Disorders (26) diagnostic criteria for sleep disorders. (3) PSQI score ≥ 8 [referring to the criteria proposed by Nofzinger et al. (27) in the American Journal of Psychiatry]. (4) The National Institutes of Health Stroke Scale (28) was used to evaluate the severity of stroke, and patients with a score ≤ 15 points with mild disease were selected. (5) Participants were aged 18–75 years, right-handed [assessed by the Edinburgh Handedness Scale (29)], and Han Chinese.

The exclusion criteria for the PSI group were as follows: (1) History of insomnia before the stroke occurrence. (2) Cerebral infarcts involving the cerebral cortex or cerebellar hemispheres; large cerebral infarcts across cerebral lobes; and the presence of moderate or severe stenosis of the cervical or intracranial large vessels. (3) Hearing impairment or communication problems or claustrophobia and unable to complete the MRI examination. (4) Presence of contraindications to MR scannings, such as metallic foreign bodies that cannot be removed from the body, magnetic stents, and cardiac pacemakers. (5) History of psychiatric diseases that severely affect brain cognitive function; or the presence of overt dementia symptoms. (6) History of brain tumor, intracranial infection or trauma (contusions, intracerebral hemorrhage, encephalomalacia, subdural hematochezia, etc.), and congenital cranial dysplasia. (7) History of alcohol dependence or other dependence on psychotropic drugs.

The inclusion criteria for the HCs were as follows: (1) participants were aged 18–75 years, right-handed; (2) no insomnia symptoms or history of insomnia, and (3) no history of intake of sedative drugs in the past 6 months. The exclusion criteria were the same as that of the PSI group, from exclusion criteria (3–7).

All included participants were informed regarding the purpose, methods, and precautions of the trial before it commenced, and they signed an informed consent form prior to participation.

### fMRI data acquisition

All participants underwent the acquisition of functional and structural images of the brain in the resting state with a Siemens Magnetom Prisma 3T MRI scanner (Siemens, Erlangen, Germany), using a 64-channel head coil. The participants were instructed to close their eyes but remain awake during the functional scans. The three-dimensional (3D)-T1-weighted (T1W) structural image parameters were as follows: TR/TE = 2,530/2.98 ms, flip angle = 8°, slice thickness = 1 mm, no gap, matrix = 512 × 512, and field of view = 256 × 256 mm. A whole brain scan was performed parallel to the midsagittal plane with 192 scanned slices. Resting-state BOLD fMRI data were acquired using a gradient echo planar pulse sequence with Simultaneous Multi-slice parallel acquisition from Siemens Prisma to achieve high temporal resolution with TR = 500 ms, TE = 30 ms, slice thickness = 3.5 mm, no gap, field of view = 224 × 224 mm, matrix = 64 × 64, flip angle = 60°, 35 axial slices, acquisition time points of 960, and acquisition time of 8 min and 45 s.

### Data pre-processing

Pre-processing of the resting-state fMRI data was performed using the Data Processing and Analysis for (Resting-State) Brain Imaging software package (version 5.0, DPABI, http://rfmri.org/DPABI) based on the MATLAB platform. The pre-processing steps included: data conversion from the DICOM format to the NifTi format and removal of image data of the first 10 time points; head motion correction (excluding participants with a displacement exceeding 3 mm in 3D space and rotation angle exceeding 3°); spatial normalization (resampling parameter 3 × 3 × 3 mm), smoothing [ReHo was first analyzed and smoothed, and ALFF and fractional ALFF (fALFF) were first smoothed and then analyzed with a 6-mm half-height bandwidth]; and removal of linear drift and low-frequency filtering (0.01–0.1 Hz bandwidth). Multiple linear regression analysis was used to reduce the effects of the white matter, cerebrospinal fluid signal, and head motion (six head motion parameters in head motion correction as covariates) on data.

### Elimination of data

During the data pre-processing, three patients with PSI were excluded because of excessive head motion (exceeding 3 mm or 3° of angular motion relative to the first volume). One patient with PSI had artifacts localized to the parietal lobe on structural T1W images which was eliminated to avoid compromising the registration of functional images with structural images. Ultimately, 23 patients with PSI were analyzed.

### Data analysis

Data analysis of ReHo, ALFF, and fALFF was performed using DPARSF (V5.2) in the DPABI software, and the analysis steps were referenced from the method proposed by Zang (30, 31). ReHo analysis was used to compute Kendall's coefficient of concordance (KCC) between a given voxel in a time series and its nearest 26 neighboring voxels. To reduce the influence of individual differences on the KCC values, we normalized the ReHo maps by dividing the KCC between each voxel by the average KCC of the whole brain. The resulting data were finally subjected to Gaussian space smoothing using a half-height bandwidth of 6 mm to reduce the effect of noise and anatomical differences in the results. Each voxel-filtered time series of the participant's brain was converted into a spectrum using a fast Fourier transform, and the power spectra were obtained; the square root of the power spectrum between 0.01 and 0.08 Hz was calculated and taken as ALFF, and ALFF at each voxel was divided by the global mean ALFF value to obtain a standardized ALFF for subsequent statistical analysis. fALFF is the ratio obtained by dividing the power at low frequencies by the power at full-frequency.

### Statistical analysis

We used the Resting-State fMRI Data Analysis Toolkit (REST, Song Xiaowei, http://www.restfmri.net/forum/) to perform two-sample *t*-tests on two groups of ReHo, ALFF, and fALFF images after individual normalization. Age, sex, years of education, and head motion parameters of the two groups were included as covariates, setting a threshold of *p* < 0.001 for the individual voxel level and *p* < 0.05 for multiple comparisons (corrected using GRF, https://afni.nimh.nih.gov/). To explore the correlation between alterations in each of the above parameters and PSQI scores of sleep quality scales in patients with PSI, we extracted the mean ReHo, ALFF, and fALFF values of all voxels in significantly different regions separately using REST, and then performed correlation analysis between the mean values of significantly different regions and PSQI scale scores in SPSS Statistics for Windows (version 25.0, IBM Corp.). Data were evaluated for normality of distribution and homogeneity of variance, and partial correlation analysis was performed if the normal distribution and homogeneity of variance were followed; age and sex were used as covariates to determine the correlation between the two groups of data. If the normal distribution or homogeneity of variance was not satisfied, the values were obtained using the general linear model regression analysis based on age and sex for all data to be analyzed, followed by Spearman correlation analysis. Statistical significance was set at *p* < 0.05.

SPSS Statistics for Windows (version 25.0, IBM Corp.) was used to analyze the clinical data. We used the Chi-square test for sex differences between the two groups, and an independent *t*-test was used to compare the age and education level between the two groups. Statistical significance was set at *p* < 0.05.

## Results

### Demographic and clinical data

**Figure 1:**
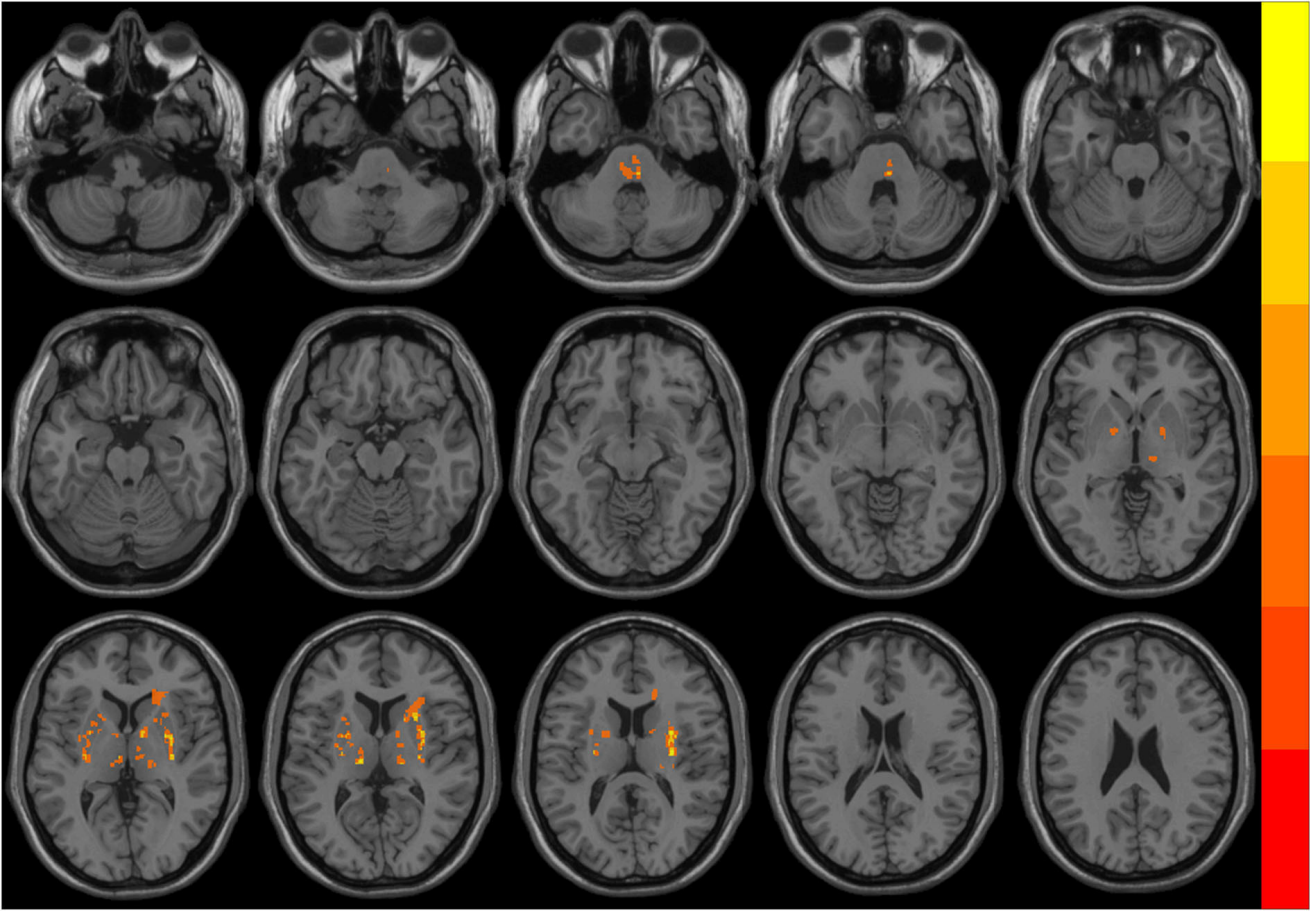
The overlapping distribution map of ischemic stroke lesions in patients with post-stroke insomnia (PSI). Different colors indicate the number of overlapping lesions at different brain regions. The infarct lesions mostly located in the pons, bilateral basal ganglia, and thalamus.

The distribution of the ischemic stroke lesions in the 23 patients with PSI after data screening was as follows: corona radiata area (7 cases), center of semiovale (seven cases), basal ganglia area (18 cases), thalamus (five cases), bridge brain (eight cases), and frontal and parietal white matter (5 cases). We delineated the regions of interest (ROIs) on the T1W structural image of all infarcts of the participants and used them as templates. All the templates of the participants were fused and superimposed. As shown in Figure 1, none of the infarct lesions involved the brain functional areas that needed to be analyzed.

A comparison of the demographics of PSI group and HCs is presented in Table 1. There were no significant differences in age (*p* = 0.732), sex (*p* = 0.555), and years of education (*p* = 0.460) between the two groups. The duration of insomnia in the patients with PSI was 3 to 36 months, with an average of 11.37 ± 6.58 months.

**Table 1:** Demographic and clinical traits of all participants.

**Characteristics**	**PSI (*n =* 23)**	**HC (*n =* 23)**	**t/*Z*/χ^2^**	***P***
Age (years)	62.48 ± 8.74	61.74 ± 5.44	−0.344	0.732
Sex (M/F)	12/11	10/13	0.348	0.555
Education (years)	11.83 ± 3.57	11.04 ± 3.66	−0.738	0.460
PSQI	14.17 ± 3.63	–		

PSQI, Pittsburgh Sleep Quality Index; PSI, post-stroke insomnia; HC, healthy controls. – means that there is no data of PSQI scale in the control group.

### Results of the ReHo analysis

**Figure 2:**
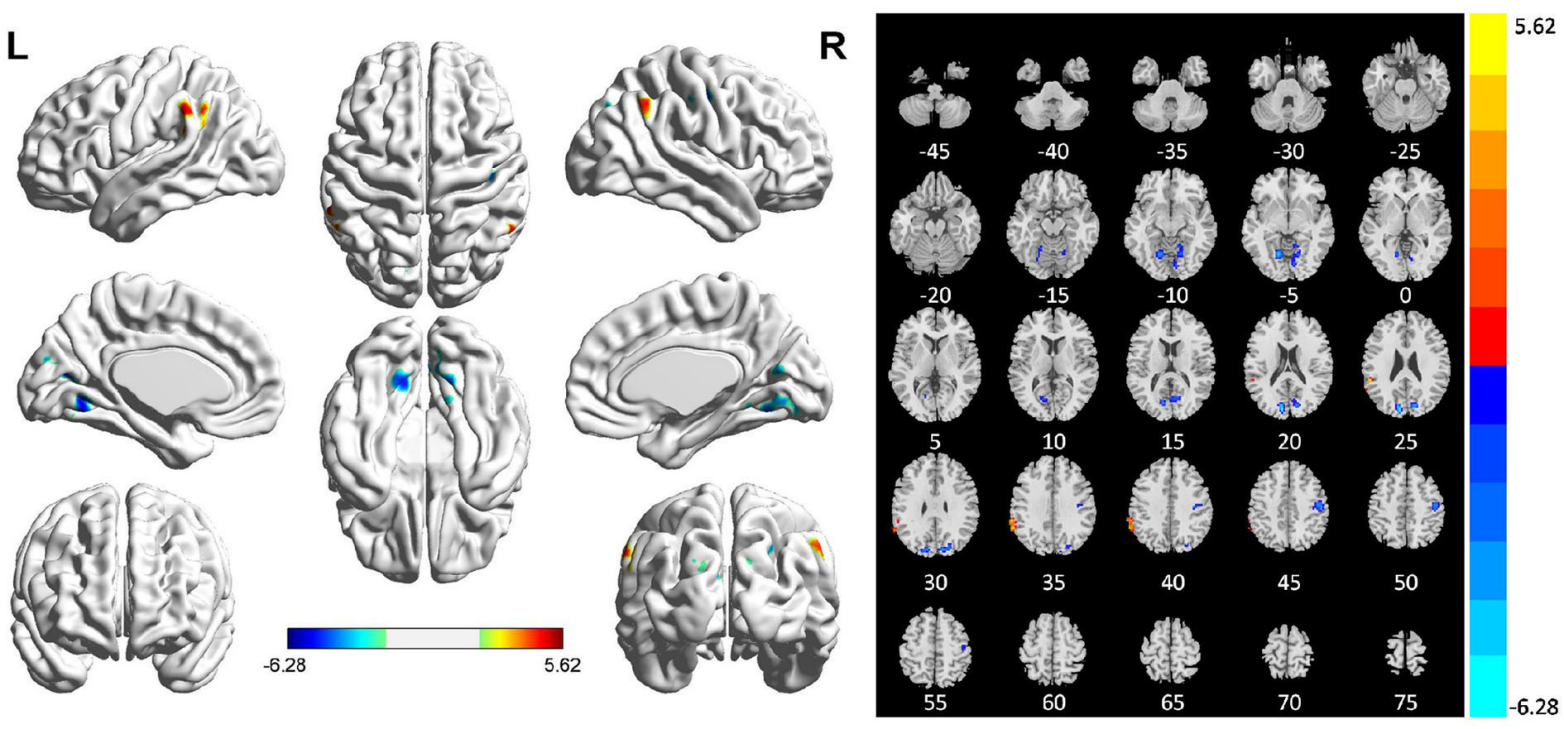
Statistically significant differences between patients with post-stroke insomnia (PSI) and healthy controls are shown in a regional homogeneity (ReHo) map of the whole-brain with magnetic resonance imaging (MRI). Patients with PSI showed a significant ReHo increase in the left supramarginal gyrus (warm colors), as well as a decrease in the bilateral lingual gyrus, right cuneus, right precentral gyrus, and posterior central gyrus with decreased ReHo values (cold colors). A T-score bar is shown below and right. Red and blue denote increases and decreases in ReHo, respectively.

Comparing the ReHo values of PSI patients and HCs, we found that the ReHo values in multiple brain regions of patients with PSI including the bilateral lingual gyrus, right cuneus, right precentral gyrus, and postcentral gyrus were significantly decreased. Furthermore, the ReHo value of PSI significantly increased in the left supramarginal gyrus (Table 2 and Figure 2).

**Table 2:** Brain regions with abnormal ReHo in patients with PSI.

**Brain regions**	**Voxels**	**Peak MNI coordinates**			***T*-value**
		***X***	***Y***	***Z***	
Cluster 1	195	−9	−87	24	−6.28
Lingual gyrus (L)	73				
Cluster 2	86	15	−63	−9	−4.55
Lingual gyrus (R)	73				
Cluster 3	120	12	−87	27	−5.11
Cuneus (R)	51				
Cluster 4	93	−63	−51	36	5.62
Supramarginal gyrus (L)	29				
Cluster 5	95	48	−15	48	−5.11
Precentral gyrus (R)	57				
Postcentral gyrus (R)	36				

ReHo, regional homogeneity; MNI, Montreal Neuroscience Institute; L, left; R, right. A positive T value indicates that the ReHo value of the PSI group was higher than that of the HCs. AAL templates were used to localize the brain regions.

### Results of the ALFF and fALFF analyses

**Figure 3:**
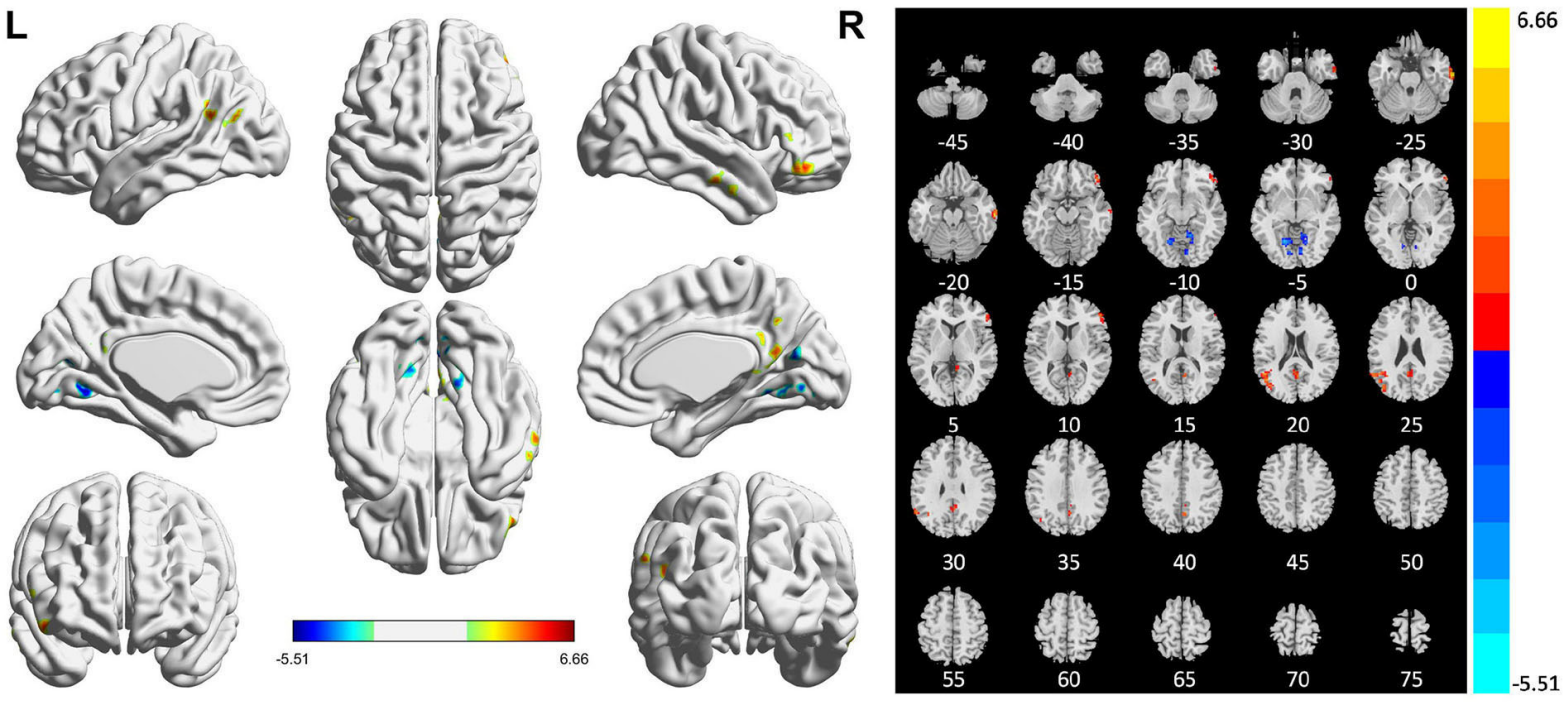
Statistically significant differences between post-stroke insomnia (PSI) patients and healthy controls are shown in the amplitude of low-frequency fluctuation (ALFF) map of the whole brain with magnetic resonance imaging (MRI). Patients with PSI showed a significant ALFF increase in the bilateral middle temporal gyrus, right inferior temporal gyrus, right inferior frontal gyrus, right limbic lobe, right precuneus, left posterior cingulate gyrus, and left middle occipital gyrus (warm colors), as well as a decrease in the bilateral lingual gyrus with decreased ALFF values (cold colors). A T-score bar is shown below and right. Red and blue denote increases and decreases in ALFF, respectively.

Compared with HCs, patients with PSI had significantly decreased ALFF values in the bilateral lingual gyrus and significantly increased ALFF values in the bilateral middle temporal gyrus, right inferior temporal gyrus, right inferior frontal gyrus (orbital and triangular parts), right limbic lobe, right precuneus, left posterior cingulate gyrus, and left middle occipital gyrus (Table 3 and Figure 3).

**Table 3:** Brain regions with abnormal ALFF in patients with PSI.

**Brain regions**	**Voxels**	**Peak MNI coordinates**			***T*-value**
		***X***	***Y***	***Z***	
Cluster 1	61	66	−12	−27	6.42
Middle temporal gyrus (R)	36				
Inferior temporal gyrus (R)	23				
Cluster 2	64	48	36	−12	6.17
Inferior frontal gyrus (orbital) (R)	36				
Inferior frontal gyrus (triangular) (R)	27				
Cluster 3	72	15	−51	−9	−5.28
Lingual gyrus(R)	57				
Cluster 4	59	−18	−60	−6	−5.51
Lingual gyrus (L)	55				
Cluster 5	74	3	−57	18	5.03
Limbic lobe (R)	52				
Precuneus (R)	31				
Posterior cingulate gyrus (L)	15				
Cluster 6	103	−60	54	27	6.66
Middle temporal gyrus (L)	35				
Middle occipital gyrus (L)	17				

ALFF, amplitude of low-frequency fluctuations; MNI, Montreal Neuroscience Institute; L, left; R, right. A positive T value indicates that the ALFF value of the PSI group is higher than that of the HCs. AAL templates were used to localize the brain regions.

**Figure 4:**
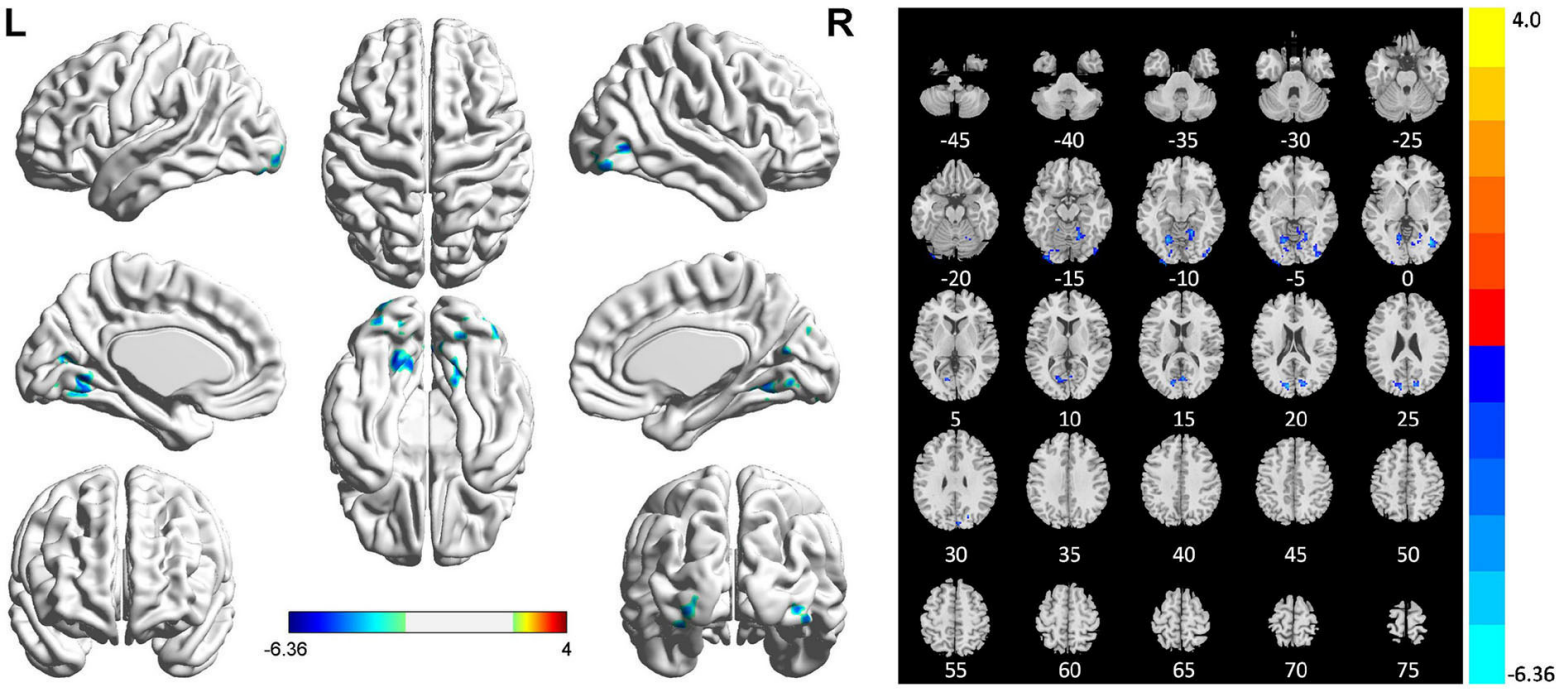
Statistically significant differences between post-stroke insomnia (PSI) patients and healthy controls are shown in the fractional amplitude of low-frequency fluctuation (fALFF) map of the whole brain with magnetic resonance imaging (MRI). Patients with PSI showed a significant decrease in fALFF in the bilateral lingual gyrus, bilateral inferior occipital gyrus, and bilateral cuneus (cold colors); no regions with increased fALFF were found. A T-score bar is shown below and right. Red and blue denote increases and decreases in fALFF, respectively.

Compared with HCs, patients with PSI had significantly decreased fALFF values in the bilateral lingual gyrus, bilateral inferior occipital gyrus, and bilateral cuneus, but no significant increase in fALFF values in any brain region (Table 4 and Figure 4).

**Table 4:** Brain regions with abnormal fALFF in patients with PSI.

**Brain regions**	**Voxels**	**Coordinate (Peak MNI**, ***X Y Z*****)**			***T*-value**
Cluster 1	63	−36	−90	−18	−5.66
Inferior occipital gyrus (L)	33				
Cluster 2	118	18	−51	−12	−5.85
Lingual gyrus (R)	75				
Cluster 3	82	39	−69	0	−6.36
Inferior occipital gyrus (R)	43				
Cluster 4	263	−9	−81	21	−5.70
Lingual gyrus (L)	102				
Cuneus (R)	37				

fALFF, fractional amplitude of low-frequency fluctuation; MNI, Montreal Neuroscience Institute; L, left; R, right. A negative T value indicates that the fALFF value of the PSI group is lower than that of the HCs. AAL templates were used to localize the brain regions.

### Correlation analysis between regional brain dysfunction and PSQI scale

**Figure 5:**
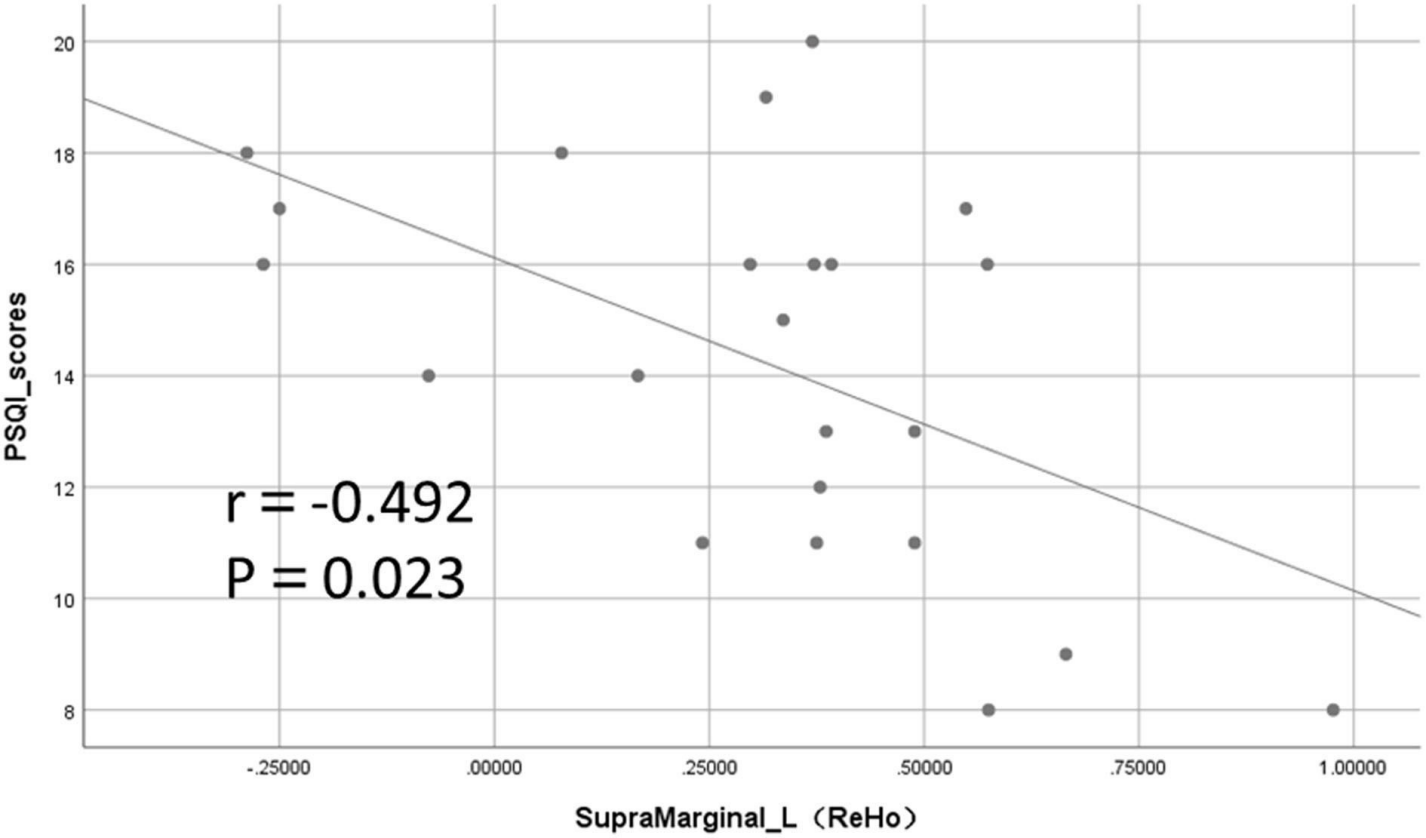
Significant negative correlation between regional homogeneity (ReHo) values and Pittsburgh Sleep Quality Index (PSQI) total score in the left supramarginal gyrus.

Among the mean values of ReHo extracted from brain regions with significant differences, the ReHo values in the left supramarginal gyrus were significantly negatively correlated with the PSQI total score (*r* = −0.492, *p* = 0.023; Figure 5). The ReHo value of the right cuneus was correlated with PSQI but not statistically different (*r* = −0.408, *p* = 0.067). However, the ReHo values of the other different brain regions and those with differences in ALFF and fALFF were not significantly correlated with the PSQI score.

## Discussion

This study showed that compared with HCs, patients with PSI had regional brain dysfunction in multiple brain regions, and comprehensive ReHo, ALFF, and fALFF results revealed that the significantly different brain regions mainly included the bilateral lingual gyrus, bilateral cuneus/precuneus, left posterior cingulate gyrus, left supramarginal gyrus, right precentral and postcentral gyrus, right frontal and temporal lobes (including inferior frontal gyrus, middle temporal gyrus, and inferior temporal gyrus), and right occipital lobe (including the superior and inferior occipital gyri). From the functional divisions, these brain regions were mainly distributed in the visual processing-related cortex (e.g., lingual gyrus, middle temporal gyrus, and superior and inferior occipital gyrus), sensorimotor cortex (e.g., right precentral and postcentral gyrus), and some default mode network (DMN) (32) brain regions (e.g., cuneus/precuneus and posterior cingulate gyrus), suggesting that the mechanism of insomnia in patients with PSI might be associated with abnormalities in brain functions, such as visual processing, sensorimotor, and DMN.

### Visual cortex dysfunction in patients with PSI

In this study, we found that the bilateral lingual gyrus showed a significant decrease in the ReHo, ALFF, and fALFF values, and the right inferior occipital gyrus also showed a significant decrease in the fALFF values. Previous studies (33–37) have shown that both the occipital lobes and lingual gyrus belong to the visual center, and the lingual gyrus has an important role in visual judgment and processing, which is mainly involved in visual information processing, and in preserving visual working memory information and working consolidation. Recent studies (38, 39) have argued that the dysfunction of the visual cortical neurons may also be an important factor in primary insomnia. Several studies (40–43) have shown some degree of brain dysfunction in the visual central brain regions such as the lingual gyrus or occipital lobe, including migraines, anxiety symptoms, apnea syndrome, and cognitive impairment in patients with Parkinson's disease and hepatic encephalopathy, also indicating that the visual cortex is associated with cognitive and anxiety states. In a study of children with obstructive sleep apnea (44), the patient group showed significantly decreased ReHo values in the right lingual gyrus and left precuneus. The ReHo values in the right lingual gyrus were negatively correlated with verbal intelligence quotient (IQ) and full-scale IQ on the Wechsler Adult Intelligence Scale, indicating that the lingual gyrus also has some correlation with cognitive performance. In a study that employed visual picture stimuli for arousal (45), the precentral cortex and cingulate cortex of patients with psychophysiological insomnia exhibited higher BOLD activation to the stimuli, which was an overreaction; the overreaction of these brain regions tended to normalize after treatment by cognitive-behavioral therapy, indicating that the stimulation of the visual cortex in patients with insomnia is important for its return to normalcy.

Our results showing reduced brain activity in the bilateral lingual gyrus and right inferior occipital gyrus are consistent with previous studies on brain function in patients with primary insomnia, which may be explained by visual cortex-related cognitive decline and abnormal emotion regulation in patients with PSI. Based on the present and previous studies, we suggest that insomnia in patients with PSI causes a persistent reduction in visual cortical activity and, consequently, impaired cognition and anxiety.

### DMN dysfunction in patients with PSI

In this study, we found that the ALFF values increased in the right precuneus, right limbic lobe, and left posterior cingulate gyrus in patients with PSI. According to previous studies (46), both the precuneus and the posterior cingulate gyrus are important to brain regions in the DMN, and their connections to structures such as the hippocampus are important and sensitive to arousal mechanisms. The DMN is associated with excessive arousal of brain regions, and under normal physiological conditions, the neural activity of the DMN is more active during the awake resting state than during the task state but is shifted from the awake resting state to sleep, particularly during deep sleep, where functional activity in multiple brain regions is significantly diminished. Previous studies (47, 48) have also shown that the DMN of patients with primary insomnia disorder remains active during sleep. The DMN of patients with psychophysiological insomnia shows an exaggerated arousal response to sleep-related stimuli, and this overreaction can be reduced by effective behavioral treatments (45).

Previous studies (49) have argued that the precuneus is critically involved in episodic memory, emotion regulation, and self-thinking. The precuneus has been shown to play a central role in several highly integrated tasks, including visuospatial image processing, episodic memory retrieval, and self-regulation processing (50). Zhao et al. (51) analyzed the ALFF between patients with primary insomnia and normal controls and found a significant increase in the ALFF values in the right precuneus, which in turn showed a significant decrease after auricular vagal stimulation of patients with insomnia, which is believed to regulate the spontaneous activity of precuneus neurons. Inhibiting introspection and improving excessive arousal in the cerebral cortex of patients may be a key mechanism for stimulating the vagus nerve to treat primary insomnia. In this study, we also found that the ALFF value in the right precuneus was significantly higher in patients with PSI. Thus, we believe that excessive arousal of the DMN is one of the reasons for insomnia in patients with PSI.

### Sensorimotor cortex dysfunction in patients with PSI

In this study, we found that ReHo values in the right precentral and postcentral gyri were significantly lower in patients with PSI than in HCs. The precentral and postcentral gyri belong to the sensorimotor cortex, which sends out nerve fibers to control the voluntary movement of skeletal muscles and to receive various sensations from the soma, including temperature and pain. Abnormalities in the sensorimotor cortex have also been mentioned in previous functional neuroimaging studies of patients with primary insomnia. Huang et al. (52) found increased FC between the sensorimotor cortex, premotor cortex, and amygdala in patients with primary insomnia compared with healthy controls, and the authors suggested that this increase in FC indicates a compensatory mechanism to overcome the negative effects of insufficient sleep. Killgore et al. (38) found enhanced FC between the primary sensory areas and the supplementary motor cortex in patients with insomnia who had difficulty falling asleep, whereas this result was not observed in those with insomnia who had difficulty maintaining their sleep state. These studies considered an increased functional activity in the sensorimotor cortex as a compensatory mechanism, which is less consistent with our findings; this may be due to different study participants leading to different results since stroke patients have multiple sites of infarct lesions. First, although we selected patients with lacunar infarcts to avoid the effects of corticospinal tract damage on the functional activity of the corresponding cortex, this may not be completely avoidable. Second, the compensatory mechanisms mentioned in these studies on primary insomnia may be insufficient to compensate for the decline in the sensorimotor cortex function caused by brain damage at the infarct lesion. Third, most fMRI studies of patients with insomnia disorder do not consider the duration of insomnia symptoms, and it is possible that, among patients with a longer duration, compensatory mechanisms in the sensorimotor cortex slowly weaken, eventually entering the decompensation phase, which leads to reduced functional activity in the precentral and postcentral gyri cortices.

### The left supramarginal gyrus dysfunction in patients with PSI

Our study showed that the ReHo value of the left supramarginal gyrus was significantly increased and negatively correlated with the PSQI score. As a part of the somatosensory associative cortex in the brain, the supramarginal gyrus is an important functional node that plays an important role in touch, spatial and limb position perception, vision, reading, and language (53–55). One study (56) also suggested that the supramarginal gyrus is involved in emotional control, egocentricity, and episodic memory. Another study (57) in patients with chronic insomnia has shown that the FC between the left supramarginal gyrus and the left middle frontal gyrus increases, suggesting that patients with chronic insomnia have an overreaction to tactile, visual, and auditory stimuli; and this suggests that the hyperarousal state of the posterior sensory cortex in the locus coeruleus noradrenergic system is crucial for chronic insomnia disorder patients in the modulation of emotions and the sleep/wake cycle. Our results showed that the ReHo value in the left supramarginal gyrus was significantly increased, which may also be related to the hyperarousal state caused by the hypersensitivity of auditory and visual perception in patients with PSI.

### Limitations

Our study correlates with several limitations. First, a relatively small sample size in this study may reduce the statistical power and the reproducibility of a study, we will continue to recruit more patients in further work. Second, the patients with PSI have high heterogeneity because of their different duration and severity of stroke. Some patients may also complain of hypertension or type 2 diabetes, which exist a potential influence on the quality of sleep, therefore, affected the assessment of insomnia. Third, the cognitive and emotional scales were not assessed in patients and the PSQI scales were not assessed in the HCs, we plan to include the screening of cognitive behaviors, anxiety, and depression in the enrolled patients with PSI in further study. More replicated research with a larger sample size is needed to confirm our study results.

## Conclusion

In general, by analyzing the regional brain functions of patients with PSI using rs-fMRI, we found that they have abnormal local activities in multiple brain regions, including the visual processing-related cortex, sensorimotor cortex, and some DMN regions. Over-arousal of the DMN and over-sensitivity of audiovisual stimuli in patients with PSI may be the main mechanisms of insomnia and can lead to a decline in cognitive function and abnormalities in emotion regulation simultaneously. The significant reduction in regional functional activity in the sensorimotor cortex in patients with PSI may be associated with brain damage in stroke, and this needs to be confirmed by further studies.

## Data availability statement

The raw data supporting the conclusions of this article will be made available by the authors, without undue reservation.

## Ethics statement

Written informed consent was obtained from the individual(s) for the publication of any potentially identifiable images or data included in this article.

## Author contributions

HW, YH, and SQ: study concepts and study design. HY and DX: data acquisition. ML, JA, and SQ: quality control of data. ML, HW, and YH: data analysis and interpretation and manuscript preparation. JA and DX: statistical analysis. HW, YH, ML, HY, JA, XL, DX, and SQ: manuscript editing and reviewing. All authors read and approved the final manuscript.

## Funding

This research received funding from the Key International Cooperation Project of National Natural Science Foundation of China (81920108019) as well as support from the Innovation and Strengthening Project of the First Affiliated Hospital of Guangzhou University of Traditional Chinese Medicine (2019QN25).

## Conflict of interest

The authors declare that the research was conducted in the absence of any commercial or financial relationships that could be construed as a potential conflict of interest.

## Publisher's note

All claims expressed in this article are solely those of the authors and do not necessarily represent those of their affiliated organizations, or those of the publisher, the editors and the reviewers. Any product that may be evaluated in this article, or claim that may be made by its manufacturer, is not guaranteed or endorsed by the publisher.
